# Bilateral inguinal transabdominal pre-peritoneal laparoscopic hernioplasty associated to bilateral laparoscopic varicocelectomy in the same intervention: a feasibility study

**DOI:** 10.1590/0100-6991e-20233468-en

**Published:** 2023-03-13

**Authors:** MIKHAEL BELKOVSKY, EDGAR OLIVEIRA SARMENTO, LUIS FELIPE COUTO NOVAES, CARLO CAMARGO PASSEROTTI, JOSÉ PONTES, LINDA FERREIRA MAXIMIANO, JOSÉ PINHATA OTOCH, JOSE ARNALDO SHIOMI DA-CRUZ

**Affiliations:** 1 - Faculdade de Medicina da USP, Técnica Cirúrgica e Cirurgia Experimental - Sâo Paulo - SP - Brasil; 2 - Hospital Alemão Oswaldo Cruz, Centro de Cirurgia Robótica - São Paulo - SP - Brasil

**Keywords:** Hernia, Inguinal, Herniorrhaphy, Varicocele, Laparoscopy, Hérnia Inguinal, Herniorrafia, Varicocele, Laparoscopia

## Abstract

**Introduction:**

Inguinal hernia and varicocele are common conditions in male population. Laparoscopy brings the opportunity to treat them simultaneously, through the same incision. However, there are different opinions about the risks for testicular perfusion of multiple procedures in the inguinal region. In this study, we assessed the feasibility of simultaneous laparoscopic procedures by studying clinical and surgical outcomes of patients undergoing bilateral inguinal hernioplasty using the transabdominal preperitoneal (TAPP) technique with and without concomitant bilateral laparoscopic varicocelectomy (VLB).

**Methods:**

a sample of 20 patients from the University Hospital of USP-SP with indirect inguinal hernia and varicocele with indication for surgical correction was selected. Patients were randomized into two groups, 10 undergoing TAPP (Group I) and 10 undergoing simultaneous TAPP and VLB (Group II). Data regarding total operative time, complications and postoperative pain was gathered and analyzed.

**Results:**

there was no statistical difference between groups regarding total operative time and postoperative pain. Only one complication (spermatic cord hematoma) was observed in Group I and no complications were observed in Group II.

**Conclusions:**

simultaneous TAPP and VLB in was shown to be effective and safe, which provides a basis for conducting studies on larger scales.

## RESUMO

**Introdução:**

hérnia inguinal e varicocele são doenças comuns na população masculina. O advento da laparoscopia traz a oportunidade de tratá-las simultaneamente, pelo mesmo acesso. Entretanto, existem divergências sobre os riscos para a perfusão testicular de múltiplos procedimentos na região inguinal. Neste estudo, avaliamos a viabilidade de procedimentos laparoscópicos simultâneos estudando resultados clínicos e cirúrgicos de pacientes submetidos à hernioplastia inguinal bilateral pela técnica transabdominal préperitoneal (TAPP) com e sem varicocelectomia laparoscópica bilateral (VLB) concomitante.

**Métodos:**

uma amostra de 20 pacientes do Hospital Universitário da USP-SP com hérnia inguinal indireta e varicocele com indicação de correção cirúrgica foi selecionada. Os pacientes foram randomizados em dois grupos, sendo 10 submetidos à TAPP (Grupo I) e 10 submetidos à TAPP e VLB simultâneas (Grupo II). O tempo operatório total, complicações e dor pós-operatória foram coletados e analisados.

**Resultados:**

não houve diferença estatística entre os grupos com relação ao tempo operatório total e à dor pós-operatória. Apenas uma complicação (hematoma do cordão espermático) foi observada no Grupo I e não foram observadas complicações no Grupo II.

**Conclusões:**

no mesmo procedimento, submeter pacientes à TAPP e à VLB no mesmo procedimento se mostrou eficaz e seguro, o que fornece embasamento para a realização de estudos em maiores escalas.

## INTRODUCTION

Varicocele is the dilation of the pampiniform plexus veins and is the main cause of infertility in men, with a prevalence of 35% to 40% in infertile men and 15% to 20% in the general male population^1^ ^,^ ^2^.

Varicocele has a complex pathophysiology. It is associated with increased testicular venous pressure, ultrastructural changes in spermatic veins, and changes in the synthesis of cytokines and testosterone^3^ ^,^ ^4^, which are related to decreased fertility.

There is a positive association between hernia and varicocele. It is believed that abdominal wall deformities in patients with hernia may cause compression of the testicular veins. Furthermore, enzymatic and biochemical disorders (such as collagen alterations) may promote the development of both diseases^5^ ^,^ ^6^. On the other hand, obesity, known to be a predisposing factor for inguinal hernia, is associated with a lower prevalence of varicocele, possibly because increased preperitoneal fat would attenuate the nutcracker phenomenon^7^.

Several approaches have been described for varicocele repair: high retroperitoneal, conventional inguinal, inguinal or subinguinal microsurgical, laparoscopic, and radiographic embolization^8^ ^,^ ^9^.

One of the most popular approaches to varicocele correction is laparoscopic varicocelectomy. It has a low incidence of complications and results in a significant improvement in symptoms, semen analysis, and fertility^10^ ^-^ ^13^.

Inguinal hernia repair is the most frequently performed procedure in general surgery^14^. Despite the Lichtenstein technique being one of the most well-established, there is still controversy regarding the best surgical approach for this pathology, especially with the arrival of new laparoscopic and microsurgical procedures^15^ ^-^ ^18^.

One of the main benefits sought by the new techniques is the reduction of postoperative pain. The microsurgical approach theoretically reduces pain by facilitating the preservation of nerve fibers, but laparoscopy generates less distension and tissue damage, completely avoiding cutaneous innervation^19^.

One of the most popular laparoscopic approaches is the transabdominal preperitoneal (TAPP). Recently, a meta-analysis of 8 studies^20^ observed that TAPP presents operative time and incidence of complications comparable to the Lichtenstein technique, and less postoperative pain, lower incidence of chronic pain (pain for more than three months after the procedure), and earlier return to work^21^.

In addition, the exposure of the peritoneal cavity that occurs in TAPP hernioplasty allows other simultaneous procedures, such as surgical correction of varicocele.

The relationship between multiple procedures in the inguinal region and the possible risks related to testicular perfusion is poorly studied^22^. Our study is the first to perform simultaneous laparoscopic varicocelectomy with TAPP hernioplasty, which seems to be a very promising approach for patients with both comorbidities.

Our objective is to analyze the feasibility and safety of these procedures in the same surgical time.

## METHODS

In this study, we prospectively analyzed 20 male patients with bilateral indirect inguinal hernia (Nyhus type 2) scheduled for surgical repair. Patients were divided into two groups: Group 1, 10 patients without varicocele, and Group 2, 10 patients with varicocele with indication for surgical correction. All patients with varicocele had bilateral disease and typical pain and underwent preoperative semen analysis, two of which showed oligoasthenospermia.

Patients in Group 1 underwent TAPP bilaterally and patients in Group 2 underwent TAPP bilaterally and bilateral laparoscopic varicocelectomy at the same surgical time.

### Surgical procedure

Throughout the procedure, the patient underwent general anesthesia at the beginning and infiltration of the trocar incisions with ropivacaine at the end. The patient was placed in horizontal dorsal decubitus, slightly inclined in a Trendelenburg position. A periumbilical incision was made to inflate the pneumoperitoneum and position a 10mm trocar. Another two 5mm trocars were positioned approximately 8cm laterally to the optics trocar.

TAPP hernioplasty was performed with incision of the peritoneum, dissection of the Bogros and Retzius spaces, identification and dissection of the hernia sac, implantation of a 15 x 12cm Prolene™ mesh (with bilateral coverage), and fixation with staples, using the Securestrap™ device.

In Group 2, before the hernioplasty, a varicocelectomy was performed, with ligation of the testicular veins with 2.0 cotton thread. The peritoneal flap was closed with continuous 3.0 Vicryl™ suture. Removal of the trocars took place under direct vision.

### Data collection

We collected and analyzed quantitative and qualitative data: patient age, total operative time in minutes (TOT), postoperative pain (POP), and complications on the 1^st^, 7^th^, 30^th^, and 180^th^ postoperative days. POP was assessed using a visual analogue scale from 0 to 10, 0 being described as no pain and 10 as the worst possible pain.

### Statistical analysis

The variables were tested for normality and did not show normal distribution, therefore being compared with the Mann-Whitney test, with a significance level of 5%, using the R software, version 4.2.1.

### Ethics and Financing

The study was approved by the Ethics Committee of the University Hospital of USP and was conducted without funding sources.

## RESULTS

The two groups had similar mean ages, 39.7±13.5 years in Group 1 and 36.8±4.3 years in Group 2.

We observed no statistically significant differences as for TOT (153.6±43.7 minutes in Group 1 and 138.4±30.8 minutes in Group 2), nor in postoperative pain (POP), as shown in Table 1. No patient had pain complaints after six months.

**Table 1:** Results

	Age (years)	TOT	POP day 1	POP day 7	POP day 30	POP day 180	Complications
Group 1	39± 13.5	153.6±43.7	6.9±0.5	4.4±0.5	1.2±1.5	0	1
Group 2	36.8±4.3	138.4±30.8	7.1±1.8	4.1±1.4	1.8±2.2	0	0
p-value	0.527	0.381	0.748	0.318	0.496	>0.999	0.305

During the study, only one complication occurred, a spermatic cord hematoma in Group 1. The hematoma developed after the patient lifted weight, despite medical advice restricting intense physical efforts in the postoperative period. The hematoma received conservative therapy and resolved after three months.

## DISCUSSION

Varicocele is a disease characterized by dilation of the pampiniform plexus veins, being the main cause of male infertility. It has a prevalence of 35% to 40% in infertile men and 15% to 20% in the general male population^23^. Most patients are asymptomatic, but up to 10% may have scrotal pain or discomfort^24^.

Varicocele treatment is indicated if it does not improve with conservative treatment^25^ or presence of all the following conditions: palpable varicocele, couple with known infertility, fertile partner or with potentially treatable infertility, and abnormal seminal parameters^26^.

Multiple studies have already demonstrated the safety and efficacy of bilateral laparoscopic varicocelectomy as a therapeutic option for varicocele^27^. Some even observed a lower recurrence rate (3.9% vs. 10.8%), shorter operative times, shorter hospital stays, faster recovery, and lower complication rates when compared with the open approach^28^. Furthermore, the laparoscopic approach yields better cosmetic results.

Inguinal hernia is a tissue protrusion in the inguinal region and can be classified as direct or indirect, depending on its relationship with the epigastric vessels^29^. It is estimated that up to 25% of men in the US will have an inguinal hernia at some point in their lives^30^. As discussed earlier, there is an association between varicocele and inguinal hernia, and several benefits have been seen with the laparoscopic approach to hernias.

The Lichtenstein technique is the most well-established technique for inguinal hernia repair. It is an open repair, with placement of a tension-free mesh^15^ ^-^ ^18^. The microsurgical approach is similar to Lichtenstein’s, but with microsurgical material. Despite demanding more resources, microsurgery reduces postoperative pain by promoting preservation of nerve fibers. The laparoscopic approach, in turn, causes less tissue damage than the microsurgical technique by avoiding cutaneous innervation. In addition, it relies on more accessible resources than microsurgery^19^.

One of the most popular laparoscopic approaches is TAPP (Transabdominal Preperitoneal), defined by preperitoneal mesh placement via laparoscopy. Recently, a meta-analysis of 8 studies^20^ observed that TAPP has operative time and incidence of complications comparable to the Lichtenstein technique, and less postoperative pain, lower incidence of chronic pain (pain for more than three months after surgery), and earlier return to work^21^.

In addition, the exposure of the peritoneal cavity that occurs in TAPP hernioplasty allows the performance of other surgeries through the same access, such as the correction of varicocele^20^.

Previously, studies were carried out on simultaneous correction of hernia and varicocele, though investigating other techniques. Chen et. al. followed 40 patients who underwent Bassini herniorrhaphy and microsurgical varicocelectomy and compared them with 20 patients who underwent herniorrhaphy exclusively. In that study, the simultaneous surgery group had only three patients with postoperative hydrocele as a complication, while the other group had no complications^8^. 

Schulster et. al. followed 141 patients who underwent microsurgical repair of inguinal hernia and varicocele in the same procedure. Despite the patients not being paired with a control group, the incidence of complications was low (only two patients had postoperative hematoma and one had varicocele recurrence)^19^. 

In our study, probably because we used different techniques from previous work, we observed even fewer complications than those in Chen’s^8^. The two groups showed similar recoveries, and after six months, no patient had symptoms and there was only one complication, a spermatic cord hematoma, which resolved with clinical treatment.

Regarding TOT, previous articles report a significant increase in mean surgical time when surgeries are performed simultaneously, both in Schulster’s study (from 102 vs. 169 minutes) and in Chen’s (38 vs. 70 minutes). In the present study, there was no difference in TOT between groups^8^ ^,^ ^19^.

The heterogeneity of surgical techniques in different studies makes it difficult to generalize conclusions, but we can state that simultaneous bilateral laparoscopic varicocelectomy and TAPP hernioplasty can be recommended for patients with hernia and varicocele. Furthermore, despite anecdotal evidence of complications^31^ ^,^ ^32^, larger studies can be performed to confirm the safety and efficacy of this joint procedure.

## CONCLUSION

Although our study observed that TAPP and varicocelectomy in the same laparoscopic approach are effective and safe, the reproducibility of these results in larger populations is crucial for the implementation of this operation in usual practice.
